# Controlling striatal function via anterior frontal cortex stimulation

**DOI:** 10.1038/s41598-018-21346-5

**Published:** 2018-02-19

**Authors:** Mieke van Holstein, Monja I. Froböse, Jacinta O’Shea, Esther Aarts, Roshan Cools

**Affiliations:** 1 Radboud University, Donders Institute for Brain, Cognition and Behavior, Nijmegen, The Netherlands; 2 Department of Psychology and Brain Research Center, University of British Columbia, Vancouver, BC Canada; 3 Wellcome Centre for Integrative Neuroimaging (WIN), Oxford Centre for Functional MRI of the Brain (FMRIB), Nuffield Department of Clinical Neurosciences, University of Oxford, John Radcliffe Hospital, Headington, Oxford, OX3 9DU UK; 4 Radboud University Medical Center, Department of Psychiatry, Nijmegen, The Netherlands

## Abstract

Motivational, cognitive and action goals are processed by distinct, topographically organized, corticostriatal circuits. We aimed to test whether processing in the striatum is under causal control by cortical regions in the human brain by investigating the effects of offline transcranial magnetic stimulation (TMS) over distinct frontal regions associated with motivational, cognitive and action goal processing. Using a three-session counterbalanced within-subject crossover design, continuous theta burst stimulation was applied over the anterior prefrontal cortex (aPFC), dorsolateral prefrontal cortex, or premotor cortex, immediately after which participants (N = 27) performed a paradigm assessing reward anticipation (motivation), task (cognitive) switching, and response (action) switching. Using task-related functional magnetic resonance imaging (fMRI), we assessed the effects of stimulation on processing in distinct regions of the striatum. To account for non-specific effects, each session consisted of a baseline (no-TMS) and a stimulation (post-TMS) fMRI run. Stimulation of the aPFC tended to decrease reward-related processing in the caudate nucleus, while stimulation of the other sites was unsuccessful. A follow-up analysis revealed that aPFC stimulation also decreased processing in the putamen as a function of the interaction between all factors (reward, cognition and action), suggesting stimulation modulated the transfer of motivational information to cortico-striatal circuitry associated with action control.

## Introduction

The frontal cortex is responsible for many higher-order functions, such as goal setting, planning, and action selection. The frontal cortical regions involved in these functions are connected with anatomically distinct sub-regions of the striatum, organized in topographically specific circuits^1–4^. Connections between ventral/anterior parts of the prefrontal cortex (PFC) and the ventral/anterior caudate nucleus form a reward circuit, and have been associated with motivational goal setting^5^. Another circuit, connecting the dorsolateral prefrontal cortex (dlPFC) with more dorsal/posterior parts of the caudate nucleus, is associated with cognitive control processes^6^. Finally, in the action circuit, (pre-)motor cortices are connected with the posterior parts of the striatum, the putamen^7^.

Initial evidence for the existence of these topographically specific cortico-striatal circuits came from work with experimental animals^8–10^. Although this animal work generally converges with human work^3,11,12^, the majority of techniques (i.e. neuroimaging) used to investigate circuits in the human brain are correlational, and thus do not inform about whether activity in one region *causes* a change in another region. Several studies have used transcranial magnetic stimulation (TMS) to show that frontal regions can exert control over striatal processing. In the absence of any specific behavioral task, offline TMS applied over the dlPFC and the motor cortex affected processing in the striatum^13–16^. Such cortical control over striatal processing was shown to be functionally specific, at least in the motor domain: TMS over cortical motor areas changed processing in the putamen during tasks that depend critically on the motor circuit^17,18^. Whether motivational reward processing in the striatum is under control of (more ventral/anterior) cortical regions in the human brain remains unclear. Here we aimed to test this, while also assessing the functional and anatomical (topographic) specificity of such fronto-striatal control.

We employed an offline TMS protocol, continuous theta burst stimulation (cTBS)^19^, aimed at decreasing^15,20,21^ neural signaling in the three frontal regions embedded within functionally distinct cortico-striatal circuits known to implement motivational, cognitive and action goal processing. Using data from an independent fMRI dataset with the same paradigm^22^, we selected three cortical stimulation sites for this study. We did so by assessing the main effect of reward, switching between tasks (cognition), and switching between response buttons (action). Thus, a reward-related region in the aPFC was selected as being part of the reward circuit, a task-switch-related region in the dlPFC as part of the cognitive circuit, and a response-switch-related region in the premotor cortex (PMC) as part of the action circuit. TMS was followed immediately by task-related blood oxygenation level dependent (BOLD) functional magnetic resonance imaging (fMRI), to measure the consequences of these interventions on the task-evoked BOLD signal in the striatal subregions embedded within each functionally distinct circuit: the ventral/anterior caudate nucleus, dorsal/posterior caudate nucleus, and the putamen. Task-related processing was assessed using an established paradigm that we have used extensively to investigate reward anticipation (reward) and cognition (switching between tasks)^22–25^. To assess task-related processing in each of these cortico-striatal circuits, we stimulated these three cortical sites on three separate days, using a counterbalanced within-subject crossover design. Moreover, to account for non-specific effects related to the day rather than to the stimulation, each session consisted of two task-related fMRI runs: a no-TMS baseline and a post-TMS stimulation run (in counterbalanced order).

Based on *in vivo* evidence about the topography of human cortico-striatal connectivity^3,12^, we predicted that TMS over each cortical target site would attenuate functionally specific activity in its striatal target site. Specifically, we predicted a) that TMS over the aPFC would attenuate activity in the ventral/anterior caudate nucleus, the main striatal target of the aPFC specifically related to one functional domain (reward-processing), but not another (task switching or response switching). We furthermore set out to test the prediction that b) TMS over the dlPFC would attenuate activity related specifically to task switching in the posterior caudate nucleus, the main striatal target of the dlPFC^3,12^ and c) that TMS over the PMC would attenuate activity related specifically to switching between response buttons in the putamen, the main striatal target of the PMC^3,12^. In addition, we anticipated these effects to be anatomically specific at the level of the striatum: i.e., we anticipated that cortical stimulation of one frontal region (e.g. TMS over the aPFC) would alter processing in a distinct region of the striatum (e.g. caudate nucleus) and not in another region (e.g. putamen). Finally, we anticipated this effect would be anatomically specific at the level of the cortex: i.e., specific to TMS over one frontal region (e.g. TMS over the aPFC, but not of the dlPFC or PMC, would alter processing in the caudate nucleus).

We performed additional exploratory analyses of the effect of TMS over the aPFC. These analyses were based on the idea that the organization of cortico-striatal circuits is not strictly parallel. Instead, signals from each cortico-striatal circuit can be transferred to other circuits^3,8–10,26^, thereby providing a mechanism by which reward-predictive signals (in the reward circuit) can engage and alter cognitive processes (in the cognitive circuit) and guide action selection (in the action circuit). We tested this possibility in two ways. First, we assessed the effect of aPFC stimulation as a function of the two-way interaction between reward anticipation and task switching, expecting modulation in more dorsal/posterior parts of the caudate nucleus, which is part of the cognitive control circuit^6,22^. Second, we assessed the effect of aPFC stimulation as a function of the three-way interaction between reward, task switching and response switching, expecting to observe modulation in the sub-region of the striatum that is part of the action circuit, namely the putamen^3,7,18^.

## Materials and Methods

### Participants

Of the 31 healthy participants who started the main experiment, 27 participants (ranging from 18–25 (mean 21.7, SD 1.95) years old; 14 men) completed all sessions and were included in the analyses (Supplementary Information). Of the 13 females, 9 used hormonal contraceptives. The menstrual cycle of the remaining 4 females was not related to the cTBS session: i.e., the aPFC, dlPFC and PMC sessions all took place on average 2.75 weeks after the last menstrual period. The experimental protocol was approved by -and the study carried out in accordance with the guidelines of -the local ethics committee (CMO Arnhem/Nijmegen: 2011/244). Participants were TMS and MRI safety screened and gave written informed consent according to the Declaration of Helsinki and the guidelines of the local ethics committee on research involving human participants. They received course credits and/or payment for their participation.

### Experimental design and procedures

All sessions took place at the Donders Centre for Cognitive Neuroimaging in Nijmegen, The Netherlands. The experiment consisted of four visits to the center: one ‘intake’ session and three experimental sessions.

#### Intake session

During the intake session, participants were introduced to the paradigm and completed two practice blocks on a laptop and a third practice block (paradigm) in the MRI scanner during the acquisition of a structural scan (MRI procedure). Next, in order to determine the stimulation intensity for each individual, we assessed their active motor threshold (aMT) (TMS procedure). Finally, participants were familiarized with the sensation of cTBS in order to ensure tolerability of cTBS over the stimulation sites (TMS procedure). After successful completion of the intake session, the first of three experimental sessions followed at least one week later.

#### Experimental sessions

**Figure 1:**
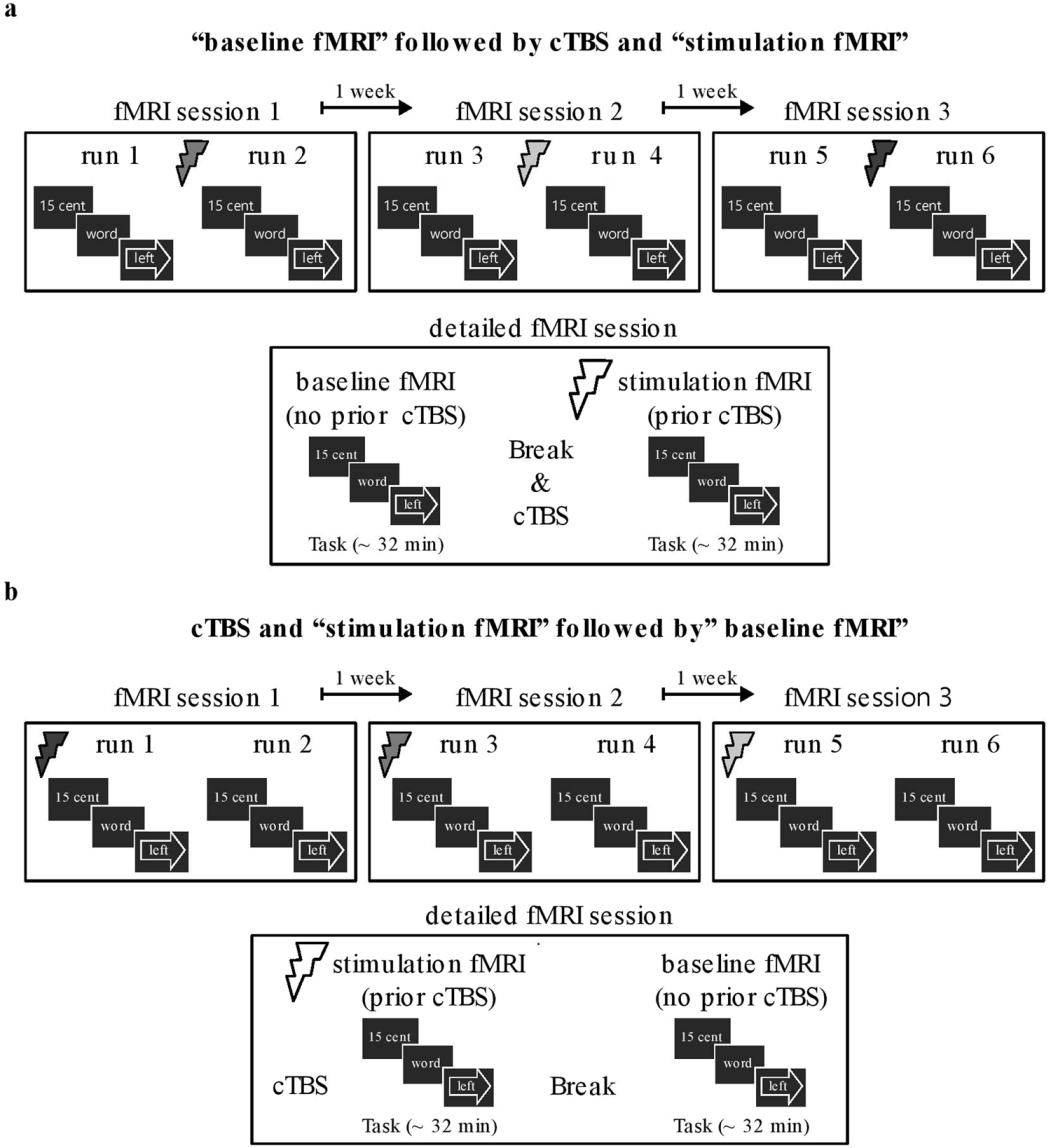
Experimental design. Each participant received continuous theta burst stimulation (cTBS) (indicated with thunderbolts) on three occasions. On each occasion, a different cortical region was targeted with TMS: anterior prefrontal cortex, dorsolateral prefrontal cortex, and dorsal premotor cortex (indicated with distinctly shaded thunderbolts in the top panels of a and b). The order in which each participant received cTBS over these regions was counterbalanced between participants. During each session, each participant completed two fMRI runs; one after TMS (stimulation fMRI) and one without prior influence of cTBS (baseline fMRI). The order in which a participant would perform the stimulation fMRI and baseline fMRI run was counterbalanced between participants (see bottom panels of a and b for a detailed illustration of an fMRI session). (**a**) Half the participants (N = 14) started with a baseline fMRI run, then received cTBS followed by the stimulation fMRI run. (**b**) The remaining participants (N = 13) received the opposite arrangement; i.e. the session started with the application of cTBS and a stimulation fMRI run, followed by a baseline fMRI run. See Table 1 for the times between cTBS and the fMRI scans.

During each experimental session, participants completed two runs in the MRI environment. To account for nonspecific effects, the paradigm was administered twice: once ~10 minutes after TMS (stimulation fMRI) and once without the prior influence of TMS (baseline fMRI)^17^. Finally, to control for order effects, we counterbalanced the order in which the stimulation fMRI and baseline fMRI runs were completed (Fig. 1). Specifically, 14 participants first performed the baseline fMRI run, followed by TMS, followed by the second fMRI run (stimulation fMRI; Fig. 1a). The remaining 13 participants started with TMS and fMRI (stimulation fMRI), followed by a 30-minute break - to allow for the TMS effects to wear off - and then the second fMRI run (baseline fMRI; Fig. 1b) (Supplementary Information: Order effects).

Previous work has shown that cTBS over the motor cortex can suppress motor evoked potential (MEP) amplitudes for up to 50 minutes after stimulation, with effects no longer evident after 60 minutes^19,21^. Therefore, in the current study, for participants who did the stimulation fMRI run first, the delay between the administration of cTBS and the start of the baseline fMRI run was approximately 90 minutes (range: 87–107 minutes) (Table 1).

**Table 1:** Time between onset of the two fMRI runs and between cTBS onset and fMRI run onset, for each session separately.

	aPFC	dlPFC	PMC	Average
**Order 1 = Baseline fMRI–cTBS–stimulation fMRI**				
cTBS–stimulation fMRI*	8.73 ± 0.43	9.11 ± 1.10	9.21 ± 1.00	9.02 ± 0.70
Baseline fMRI–stimulation fMRI*	94.14 ± 5.60	92.86 ± 4.70	92.07 ± 4.84	93.02 ± 3.44
**Order 2 = cTBS**–**stimulation fMRI–baseline fMRI**				
cTBS**–**stimulation fMRI*	10.02 ± 2.01	9.37 ± 0.90	9.47 ± 0.98	9.62 ± 1.06
Stimulation fMRI**–**baseline fMRI*	85.31 ± 11.16	83.08 ± 2.93	82.46 ± 3.26	83.62 ± 4.81
cTBS**–**baseline fMRI*	92.41 ± 5.48	92.94 ± 3.22	91.45 ± 3.13	92.26 ± 3.44

*Differences are calculated from the start of the first fMRI run (duration: ~35 minutes) or the start of 40 s cTBS, to the start of the second fMRI run (duration: ~35 minutes). Times displayed are average ± SD minutes.

### Paradigm

**Figure 2:**
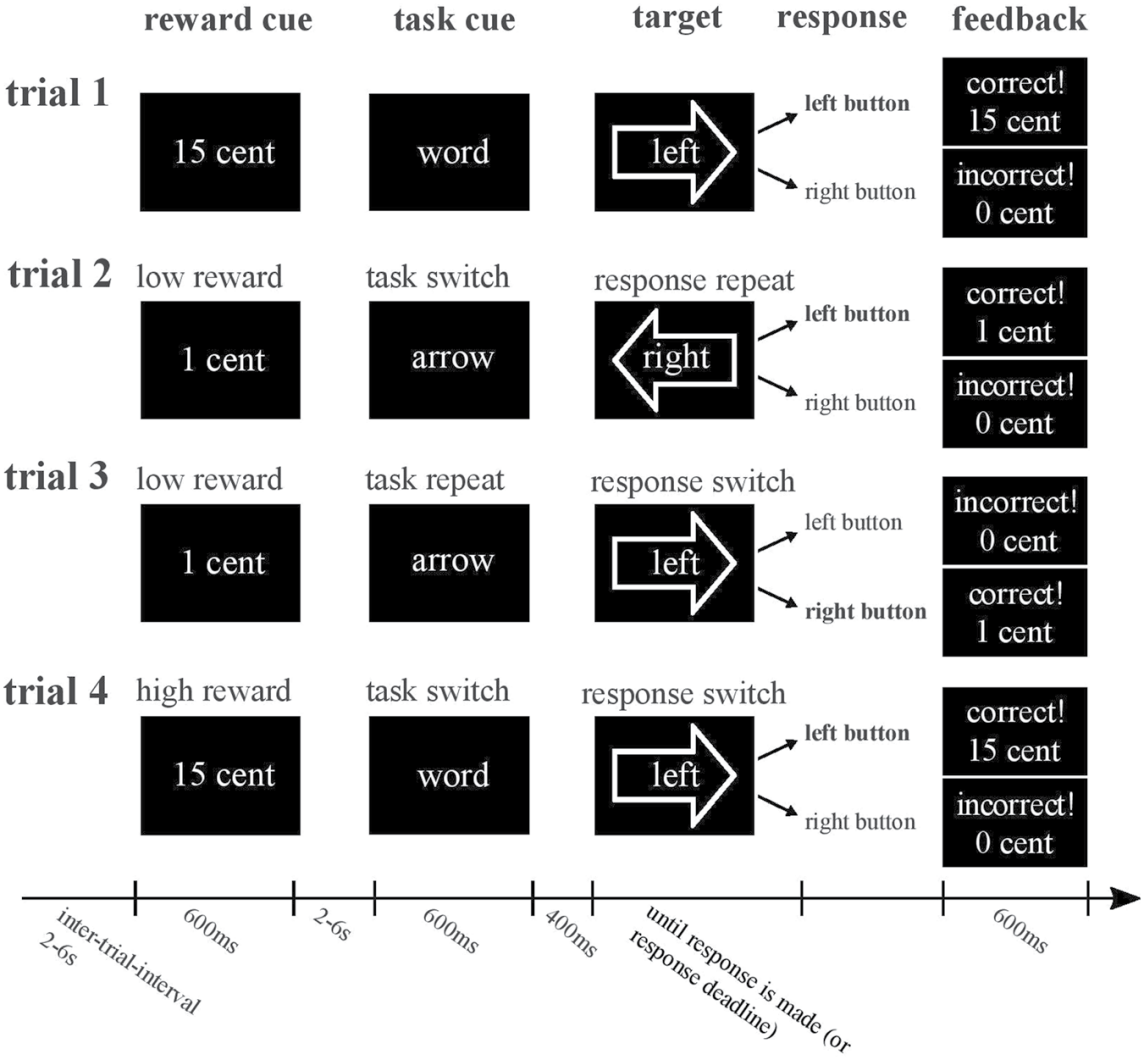
Task and response switching paradigm with reward manipulation. Participants always had to respond to response-incongruent arrow-word combinations (targets) with a left or right button press, either by responding to the direction indicated by the arrow (i.e. [←] or [→]) or to the direction indicated by the word (i.e. ‘left’ or ‘right’). A task cue preceding the target (by 400 ms) indicated which task (arrow or word) the participant had to respond to on the current trial. The task on the current trial could either change (unpredictably) with respect to the preceding trial (i.e. task switch trial; arrow-word as in trial 4, or word-arrow as in trial 2) or remain the same (i.e. task repeat trial; arrow-arrow (trial 3), or word-word). In addition to such task switches, the paradigm allowed us to look at response switches, i.e. whether the correct response (left or right button, the correct response is printed in bold), remained the same (i.e. response repeat trial; left-left as in trial 2, or right-right) or switched (i.e. response switch trial; right-left, as in trial 4 or left-right as in trial 3) compared with the previous trial. In the current version of the paradigm we made sure that the task switches occurred independently from response switches; half of the task-repeat trials and half of the task-switch trials required a switch of the response button (e.g. trial 3 and 4 respectively), whereas the remaining half of the trials required a response repetition (e.g. trial 2). In addition, as in previous versions, we manipulated the amount of anticipated reward (€0.01 vs. €0.15) on a trial-by-trial basis by means of a reward anticipation cue. At the start of each trial this reward cue indicated the amount of reward for that trial, contingent on a correct and sufficiently fast button press (see paradigm). Immediately following the response, feedback was given (e.g., “correct! 15 cents” in green ink, or “incorrect! 0 cents” in red ink) see also ref.^25^.

Participants performed a task-switching paradigm with a reward manipulation that has been used previously e.g.^25^. In the current version, minor changes were made to include a response-switching component. All details of the task are described in the legend of Fig. 2.

Each session started with a number of practice blocks (Supplementary Information). The paradigm consisted of 160 trials and lasted ~35 minutes with a 30s break every 32 trials. In the breaks and at the end of each run (i.e. after 160 trials) the cumulative amount of money the participant earned was displayed on the screen (max. €12.80). Participants were informed in advance that we would keep track of the total amount of money on each run and that the total earnings of one of the six runs would be added to their financial compensation as a bonus. Which run this was, was determined by chance at the end of the third session.

### Transcranial Magnetic Stimulation (TMS) procedure

#### Selection and targeting of stimulation sites

**Figure 3:**
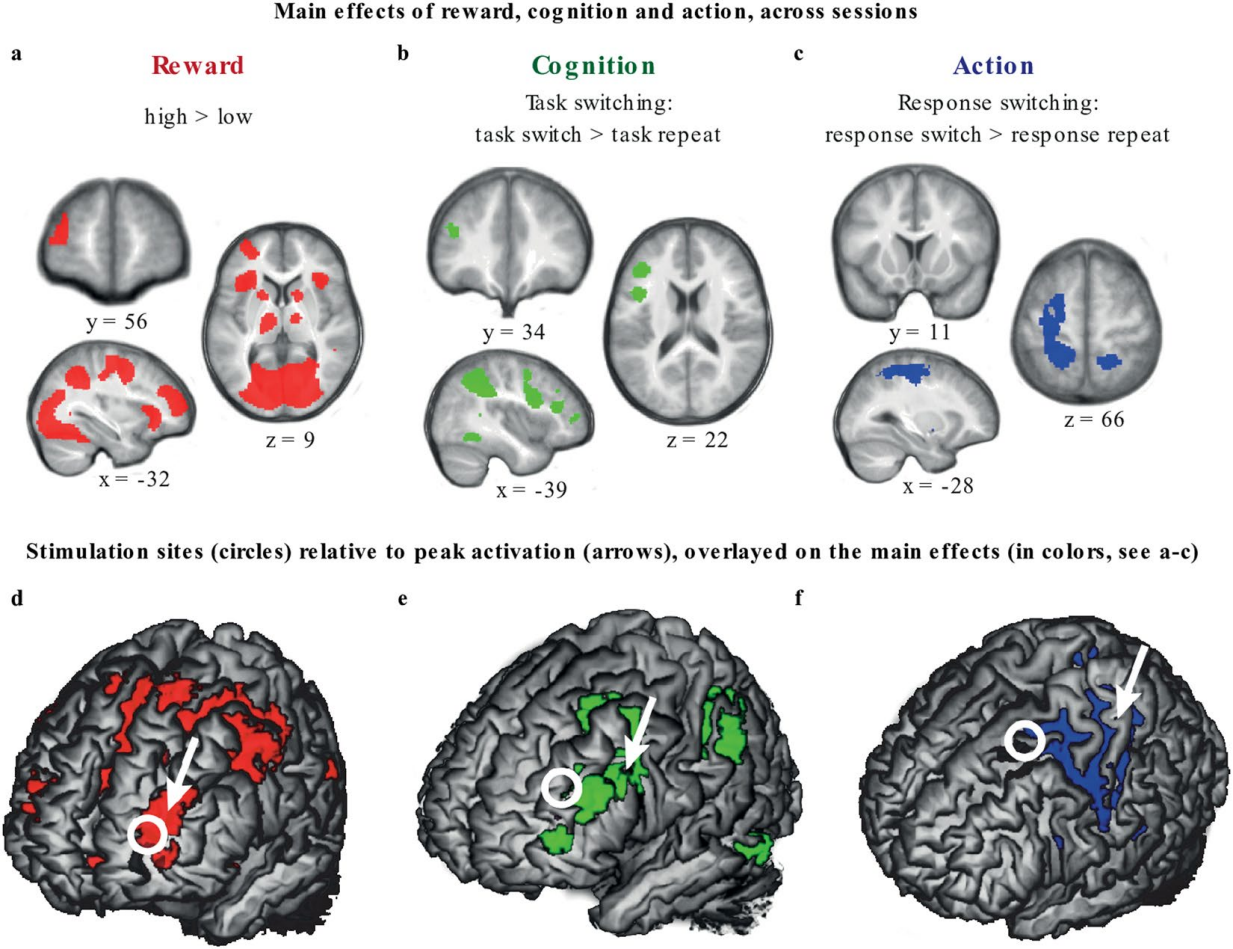
Main effects of task manipulations across runs. The three vertical panels show the main effect of: Reward anticipation (high reward >low reward; red; **a** and **d)**, task switching (task switch > task repeat; green; **b** and **e**); and response switching (response switch > response repeat; blue; **c** and **f)**, combined across all experimental runs. Results are displayed at a threshold of t = 3.14 (P_UNC_ < 0.001). See Table 2 and the main text for whole-brain and FWE small-volume corrected results. For illustration purposes, white circles (8 mm diameter) indicate the coordinates at which stimulation was applied to the **d**) anterior prefrontal cortex, **e**) dorsolateral prefrontal cortex, and **f**) premotor cortex, determined based on an independent fMRI dataset^22^. Arrows point to the location of the peak activations nearest the stimulation site for the relevant factor of interest in the current study. The rendered images show regions with a search depth of 8 mm.

The mean MNI coordinates for targeting TMS at each of the three stimulation sites were determined by assessing the peak BOLD-fMRI activations for the main effect of Reward (high > low reward cue), the main effect of Task switching (task switch > task repeat), and the main effect of Response switching (response switch > response repeat) in the frontal cortex. This was performed on a previously published fMRI dataset that had used the same behavioral paradigm^22^. A region in the left anterior PFC (aPFC: −30, 60, 8, Brodmann area 10; white circle in Fig. 3d) was identified as part of the reward network; a region in the left dorsolateral PFC (dlPFC: −36,36, 20, Brodmann area 46; white circle in Fig. 3e) was identified as part of the cognitive (task switching) network; and a region in the left premotor cortex (PMC: −28, 10, 66, Brodmann area 6; white circle in Fig. 3f) was identified as part of the action (response switching) network.

To determine the coil positioning, we converted the stimulation targets in group mean MNI coordinates into individuals’ native anatomical space (Supplementary Information). During the intake session, participants were familiarized with the sensation of cTBS over each of these regions by undergoing 10 seconds (instead of 40 seconds) of stimulation, using otherwise identical parameters to those used in the main experimental sessions (Supplementary Information).

#### Continuous Theta Burst Stimulation (cTBS) protocol

During the experimental sessions, cTBS was administered following the protocol described by Huang and colleagues^19^. They applied cTBS (bursts of three 50 Hz pulses every 200 ms for 40 s, i.e. a total of 600 pulse) over the motor cortex at 80% of the aMT and reported a depression of MEP amplitudes over a subsequent period up to 60 minutes see also ref.^21^. We chose to use this stimulation protocol (cTBS) which inhibits motor corticospinal excitability, rather than TMS (e.g. 10 Hz repetitive TMS, rTMS) - which is excitatory - for practical reasons. Although rTMS has been shown previously to modulate striatal processing, and so could have been used to address our research questions, a practical advantage of cTBS over rTMS guided our choice: When applied near facial muscles, cTBS over the aPFC is reportedly well tolerated^20^, contrary to anecdotal evidence regarding rTMS protocols.

TMS pulses (biphasic) were administered through a figure-eight coil (75 mm diameter), connected to a MagPro X100 stimulator (Mag Venture, Denmark). We used standard electromyogram (EMG) recordings to visualize MEPs from the first dorsal interosseous muscle of the right hand and to determine the resting motor threshold, using a standard protocol^20,27^ (Supplementary Information).

### Magnetic Resonance Imaging (MRI) procedure

#### MRI acquisition

MRI images were acquired on a 3-Tesla MRI system (Magnetom TrioTim; Siemens Medical Systems, Erlangen, Germany), using a 32-channel head coil. High-resolution T1-weighted MP-RAGE anatomical images were acquired during the intake session (GRAPPA acceleration factor 2; repetition time 2300 ms; echo time 3.03 ms; field of view: 256 mm; voxel size 1 mm^3^). In order to obtain a good signal-to-noise ratio for brain areas susceptible to dropout, functional images were acquired using a T2*-weighted multi-echo gradient-echo planar sequence (repetition time: 2090 ms; echo times for 4 echoes: 9.4, 21.2, 33, 45 ms; flip angle: 90°; 32 ascending slices; 0.5 mm slice gap; voxel size 3.5 × 3.5 × 3 mm)^28^. In addition, following each task-related fMRI acquisition, we acquired 266 resting state scans (data not reported).

#### Preprocessing of task-related fMRI data

All data were analyzed using SPM8 (Statistical Parametric Mapping; Wellcome Department London, UK, http://www.fil.ion.ucl.ac.uk/spm). Prior to standard preprocessing, the four echo images were combined using echo summation (Supplementary Information). The combined images were slice-time corrected to the middle slice, normalized to a standard template using the transformation matrix from the unified segmentation procedure, and smoothed (Supplementary Information).

### Statistical analyses

#### Statistical analysis of behavioral data

Behavioral analyses were performed on the response times (RTs) and error rates (Supplementary Information). Results were analyzed using a repeated measures ANOVA with the factors TMS condition (stimulation or baseline), Reward (high vs. low), Task switching (task switch vs. task repeat) and Response switching (response switch vs. response repeat). We report effect sizes for all significant effects (p < 0.05) using partial eta squared (η_p_^2^). Effect sizes larger than 0.01, 0.06 and 0.14 are considered small, medium and large, respectively^29^.

#### Statistical analysis of fMRI data

First-level analysis: The preprocessed fMRI time series were analyzed at the first level using one general linear model (GLM) for each participant, including all sessions. For each session, the following 26 task-related regressors were modeled at the onset of the stimulus (duration = 0) convolved with a canonical hemodynamic response function. The main effect of reward was modelled at the onset of the reward cue (high/low). For the main effects of task switching, response switching and the interaction between task factors, the following events were modelled at the presentation of the arrow-word target: [the type of reward trial (high/low) x the task cue (arrow/word) x task (switch/repeat) x response (switch/repeat)]. We additionally modelled the feedback (correct low/correct high/incorrect/too late), breaks (duration = 30 s), the first trial of each block, and response omissions. In addition, we accounted for residual head motion, movement-related intensity changes and low-frequency signals by including 24 motion parameters, and taking into account CSF signals based on each individuals’ segmented T1 scan to account for movement-related intensity changes and physiological noise (Supplementary Information).

To assess the main effects (Reward, Task switching, Response switching), we generated, for each participant, a contrast image at the first level. For the main effect of Reward (high >low reward cue), the effect was time-locked to the reward cue. The contrast images for the main effects of Task switching (task switch >task repeat) and Response switching (response switch >response repeat) were all time-locked to the presentation of the arrow-word target. In addition, we generated a contrast image for each interaction effect (i.e. Reward x Task switch and Reward x Task switch x Response switch) time-locked to the presentation of the arrow-word target.

Second-level analysis: At the second level, the contrast images of each effect were subjected to a full factorial GLM, including all sessions of each participant. First, we assessed the main effect of each component of the paradigm (i.e. Reward, Task switching and Response switching) across all six sessions (i.e. irrespective of TMS) (Fig. 3). Secondly, we addressed our primary question: whether stimulation of the prefrontal cortex *influences* task-related striatal processing. To this end, we assessed whether stimulation of the aPFC, relative to baseline (i.e. the contrast aPFC_BASE-STIM_), changed Reward-related processing in the striatum. More specifically, we anticipated that stimulation of the aPFC (vs. baseline) would reduce the BOLD response exclusively as a function of the main effect of Reward, and not as a function of the main effect of Task switching or Response switching (functional specificity), selectively in the caudate nucleus and not in the putamen (anatomical specificity at the level of the striatum) and exclusively after stimulation of the aPFC and not after stimulation of the dlPFC or PMC (anatomical specificity at the level of the cortex) (see Supplementary Information for a more detailed description of these analyses). In addition, we assessed whether stimulation of the dlPFC (dlPFC_BASE-STIM_) and the PMC (PMC_BASE-STIM_) changed processing related to Task-switching and Response-switching in the striatum. More specifically, we predicted TMS would modulate the BOLD response selectively in a more dorsal/posterior portion of the caudate nucleus and in the putamen, respectively. Finally, we performed two post-hoc exploratory analyses to further explore the effects of aPFC stimulation. Here, we aimed to test whether stimulation of the aPFC would not only affect processing as a function of Reward, but also processing as a function of the integration between Reward and Task switching, or between Reward, Task switching and Response switching. This was based on the idea that the organization of cortico-striatal circuits is not strictly parallel, but that signals from each cortico-striatal circuit can be transferred to other circuits^3,8–10,26^. To address this, we generated contrast images at the first level for a 2-way interaction (between Reward and Task switching) and a 3-way interaction (between Reward, Task switching and Response switching), all time-locked to the presentation of the arrow-word target. Functional and anatomical specificity was assessed for any effects below P_FWE_ < 0.05 (Supplementary Information).

**Figure 4:**
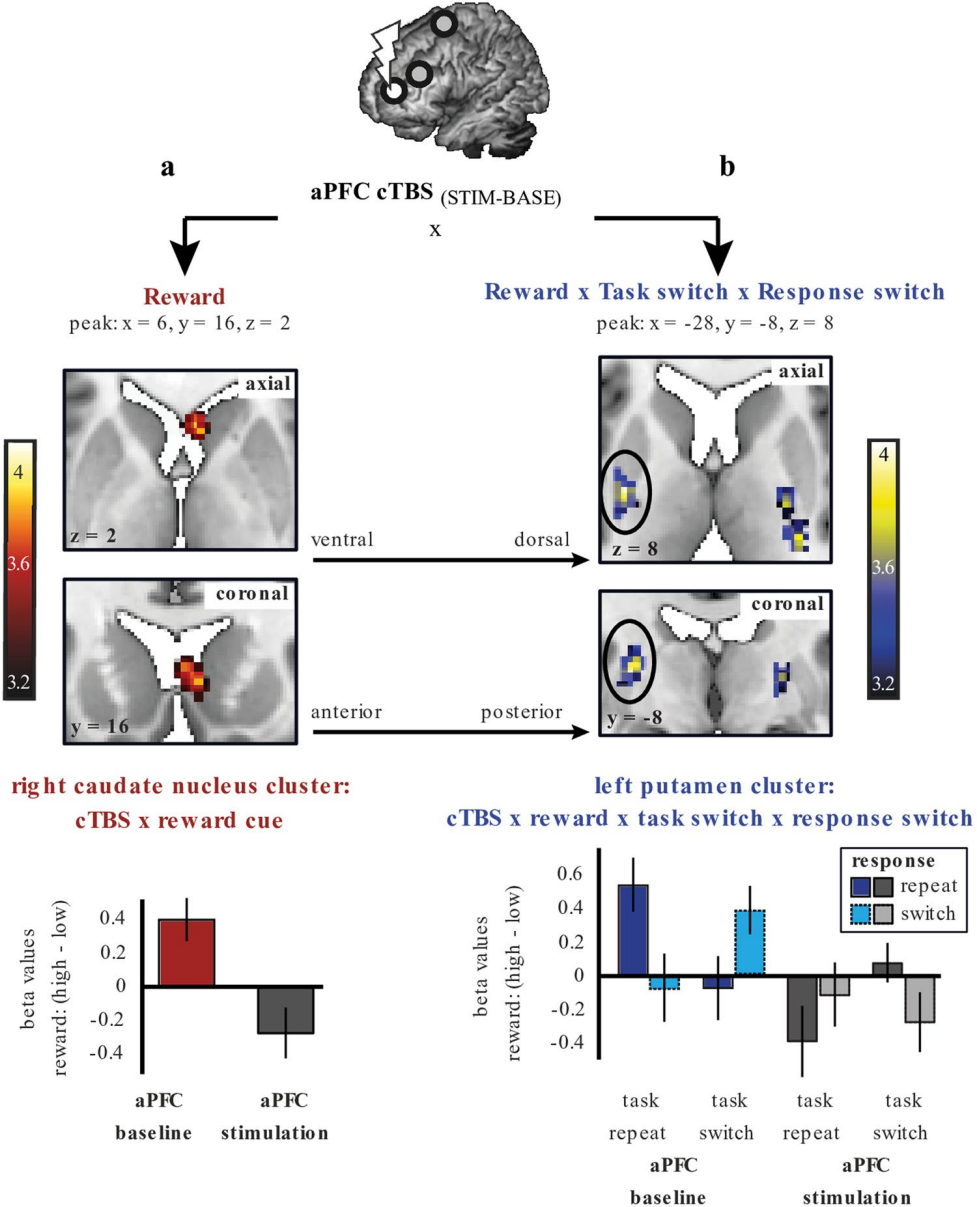
Effect of aPFC stimulation versus baseline for Reward (red) and for the interaction between Reward, Task switching and Response switching (blue). Top: Brain maps for the effect of cTBS_(baseline-stimulation)_ on the main effect of Reward (**a-** in red) and on the interaction between Reward, Task switching and Response switching (**b-** in blue) are shown at a threshold of P_UNC_ < 0.001, t > 3.14. The FWE-corrected significant result (p < 0.025 after Bonferroni correction for 2 ROIs) is indicated by the black circles. Bottom: Plots of the beta values extracted from the right caudate (**a**) and left putamen (**b**) cluster displayed in the top part of the figure. See Supplementary Figure S1 for whole-brain maps across the axial (dorsal to ventral) and coronal (anterior to posterior) plane. Color scales reflect t-values. Note that the results are displayed at a low threshold (P_UNC_ < 0.001, t > 3.14), but that statistical significance (FWE-corrected) of the results was assessed in two anatomically defined regions of interest (i.e. restricted to the grey matter of the bilateral caudate nucleus and bilateral putamen). A high-resolution default MRIcron template was used as a background.

Statistical testing and data visualization: Effects that survived a family wise error (FWE) correction (P_FWE_ < 0.05) were considered significant. We assessed effects at the whole-brain level and for specific hypotheses regarding the caudate nucleus and putamen. Therefore, we applied a small volume correction (SVC) in the bilateral caudate nucleus and bilateral putamen to assess the effects of cTBS on each of the main effects (Fig. 4). We used the AAL atlas, as implemented in WFU Pickatlas in SPM^30^, to obtain an ROI for the left and right caudate nucleus and one for the left and right putamen. The use of these two distinct ROIs allowed us to test our hypothesis regarding anatomical specificity at the level of the striatum (see Second-level analysis). To account for the two regions of interest (the caudate nucleus and the putamen), P_FWE_ effects below (0.05/2 = ) 0.025 were considered significant for the ROI analyses.

For visualization purposes, statistical maps are overlaid onto a study-specific template (Supplementary Information) and displayed at t = 3.14, p < 0.001, unless stated otherwise. Significant (FWE-corrected) results are reported in the text and tables and are indicated by black circles in Fig. 4. To visualize any effect that survived a P_FWE_ < 0.05 threshold, we extracted the beta values for the main effect of Reward and for the interaction between Reward, Task switching and Response switching from each activated cluster using the MarsBar function in SPM (Fig. 4, Supplementary Information).

### Data Availability

The datasets generated and/or analyzed during the current study are available from the corresponding author.

## Results

### Effects of task on brain activity independent of cTBS

First, we assessed the main effect of Reward_(HIGH VS. LOW)_, Task switching_(SWITCH VS. REPEAT)_, and Response switching_(SWITCH VS. REPEAT)_, independent of cTBS, by pooling the data across runs (i.e. across three stimulation fMRI and three baseline fMRI runs).

Comparing the neural signal during high versus low Reward cues revealed a large bilateral network of regions, including the striatum, lingual gyrus, thalamus, cingulate cortex and the aPFC (Table 2 and Fig. 3a). The opposite contrast_(LOW–HIGH)_ revealed three clusters in the prefrontal cortex (Table 2).

**Table 2:** Main effect of Reward anticipation (across all sessions).

	Peak MNI coordinate			Statistic		
	x	y	z	t-value	p-value (peak)	cluster size
**Reward (high > low)**						
Frontal lobe						
SFG/MFG (B10)	−32	50	18	6.15	P_FWE_ < 0.001	977
SFG: SMA (B6)^BI^, dACC (B32)^BI^	−8	4	60	8.79	P_FWE_ < 0.001	7318
Subcortical						
Striatum^BI^: caudate nucleus*	10	10	0	8.11	P_FWE_ < 0.001	4543
Thalamus	24	−24	4	4.92	P_FWE_ < 0.043	50
Occipital lobe						
Lingual gyrus (B17)^BI^	−12	−94	−4	16.18	P_FWE_ < 0.001	213500
Parietal lobe						
Posterior cingulate cortex^BI^ (B23)	−4	−30	26	5.47	P_FWE_ = 0.005	538
**Reward (low > high)**						
Frontal lobe						
IFG (B10)	46	40	2	5.22	P_FWE_ = 0.013	555
MFG: OFC (B11)	−40	36	−12	5.20	P_FWE_ = 0.015	403
MFG (B9)	32	32	48	5.07	P_FWE_ = 0.025	697

The table shows all areas that were significant at peak P_FWE_ < 0.05.

*Cluster includes the caudate nucleus, putamen, nucleus accumbens, midbrain, thalamus, pallidum and extends into the insular cortex and IFG; SMA = supplementary motor area, dACC = dorsal anterior cingulate cortex; OFC = orbitofrontal cortex; SFG: superior frontal gyrus; MFG: middle frontal gyrus; IFG: inferior frontal gyrus; BI = bilateral cluster; B = Brodmann area.

During trials on which the Task switched, compared with trials on which the Task was repeated (Task_SWITCH-REPEAT_), a network of three clusters, encompassing the inferior frontal gyrus, cingulate gyrus, superior parietal lobe, and inferior temporal gyrus was activated (Fig. 3b). More specifically, the peak of one cluster was located in the inferior frontal gyrus (P_peak_FWE_ < 0.001, cluster size = 1755, T = 6.01, peak x, y, z = −40, 4, 30), one in the parietal lobe (P_peak_FWE_ < 0.001, cluster size = 3760, T = 8.72, peak x, y, z = −24, −66, 50) and one in the temporal lobe (P_peak_FWE_ < 0.001, cluster size = 751, T = 6.48, peak x, y, z = −48, −52, −12). A region of interest analysis with focus on the signal in the striatum did not reveal any significant effects of Task switching in either the caudate nucleus or the putamen. No regions exhibited increased activation during the opposite contrast (Task_REPEAT-SWITCH_).

One large cluster was more active during Response switching compared with Response repetition trials (Response_SWITCH-REPEAT_; Fig. 3c). This left lateralized cluster included the primary motor cortex (B4), premotor cortex (B6), primary somatosensory cortex, precentral gyrus and the primary somatosensory cortex (B3) and extended posteriorly into the parietal lobe, i.e. the postcentral gyrus (P_peak_FWE_ < 0.001, t = 6.61, z = 6.19, cluster size = 3250, peak x, y, z = −40, −36, 54). A region of interest analysis with focus on the signal in the striatum did not reveal any significant effects of Response switching in either the caudate nucleus or the putamen. No regions exhibited increased activation during the opposite contrast (Response_REPEAT-SWITCH_).

In summary, analyses of the fMRI activation patterns (independent of TMS) revealed a significant main effect of Reward anticipation_(HIGH vs. LOW REWARD)_ in the caudate nucleus. However, no significant voxels were detected in the striatum as a function of the main effect of Task switching_(SWITCH–REPEAT)_ or the main effect of Response switching_(SWITCH–REPEAT)_, i.e. across all sessions. Therefore -in supplementary analyses- we investigated the data from each of the three fMRI sessions separately, to determine whether there was a main effect of Task switching during the baseline run of the dlPFC session, and a main effect of Response switching during the baseline run of the PMC session. These analyses also did not reveal any significant clusters in the striatum. The absence of effects in the striatum undermines the logic of testing whether cTBS decreases the BOLD response in the striatum during the stimulation vs. baseline run if no significant activation is detected during the baseline run. In addition, activated voxels for the main effect of Task switching were located up to 3.2 cm away from the dlPFC stimulation site, and the activated voxels for the main effect of Response switching were located up to 4.6 cm from the PMC stimulation site. By contrast, the stimulation coordinate for the aPFC was located within 1 cm of the cluster that was activated by the main effect of reward. Perhaps not surprisingly then, analysis of the effect of cTBS over the dlPFC_(BASE-STIM)_ on Task switching_(SWITCH–REPEAT)_ or of cTBS over the PMC_(BASE-STIM)_ on Response switching_(SWITCH–REPEAT)_ did not reveal any brain regions where cTBS changed the BOLD-fMRI signal. Therefore, we will focus on results obtained after stimulation of the aPFC.

In what follows, we assessed our key hypothesis for the one session where the task evoked a significant effect on the BOLD response in the striatum: the aPFC, and asked whether (1) changes in task-related processing occurred in the striatum after stimulating the aPFC, and whether these effects were (2) anatomically and (3) functionally specific.

### Effects of anterior prefrontal cortical stimulation on striatal processing

#### Functionally-specific effects of aPFC stimulation on striatal processing

Reward-related BOLD signal in the right caudate nucleus was decreased after aPFC stimulation compared with baseline (aPFC_BASE-STIM_ x Reward_HIGH-LOW_: P_SVC_FWE_ = 0.040, k = 17, T = 3.78, z = 3.69, peak x, y, z = 6, 16, 2; Fig. 4–in red). The effect was located in the anterior portion of the caudate nucleus, and there was no such effect of aPFC stimulation on reward-related signal in the putamen, or elsewhere in the brain. However, this effect did not survive the Bonferroni correction (p < 0.025) that we applied to the small volume corrected results in order to account for two striatal search volumes (bilateral caudate nucleus and putamen). Although we do not consider these results significant after correcting for multiple comparisons, for completeness, we report the assessment of functional and anatomical specificity in the Supplementary Information.

#### Transfer of task-related signal across cortico-striatal circuits

In an additional analysis we tested whether information from the Reward circuit was transferred across cortico-striatal circuits. This idea was motivated by anatomical work which has challenged the idea that cortico-striatal circuits are strictly parallel^3,8–10,26^. Instead, according to these proposals, signals from each circuit can be transferred to other circuits. We reasoned that – if these circuits are not strictly parallel - stimulation of the aPFC could affect task-related activity in more posterior parts of the striatum during the integration of Reward and Task switching or during the integration of all three factors: Reward, Task switching and Response switching.

There was no effect of aPFC stimulation on the integration of Reward and Task switching (i.e.: no 3-way interaction between aPFC_BASE-STIM_ x Reward_HIGH vs. LOW_ x Task_SWITCH vs. REPEAT_ (i.e. irrespective of Response switching). However, aPFC stimulation did modulate the integration between all three factors (i.e.: the 4-way interaction of aPFC_BASE-STIM_ x Reward_HIGH vs. LOW_ x Task_SWITCH vs. REPEAT_ x Response_SWITCH vs. REPEAT_ was significant). Assessment of the effect of aPFC stimulation on this integration (4-way interaction: aPFC_BASE-STIM_ x Reward_HIGH vs. LOW_ x Task_SWITCH vs. REPEAT_ x Response_SWITCH vs. REPEAT_) revealed that aPFC stimulation decreased signaling in the left putamen (Fig. 4b) (left: P_SVC_FWE_ = 0.020, t = 3.96, k = 66; z = 3.86, peak x, y, z = −28, −8, 8). This effect remained significant after correcting for the two search volumes (i.e., p < 0.025). The signal in the right putamen was not significant (P_SVC_UNCORR_ < 0.001, k = 1). To illustrate these effects, the whole-brain maps at an uncorrected threshold of p < 0.001 are shown in Supplementary Figure S1. To investigate further the nature of this interaction, one-sample t-tests were conducted on the Reward x Task x Response contrast images separately for each fMRI run.

Analysis of the aPFC baseline fMRI run revealed a significant Reward x Task x Response effect in the left putamen (left putamen: P_SVC_FWE_ = 0.01, t = 4.95, k = 64, z = 4.12, peak x, y, z = −30, −18, 6; right putamen: P_SVC_FWE_ = 0.086, t = 4.21, k = 13, z = 3.64, peak x, y, z = 28, −12, 10) (Fig. 4b - blue bars). This was driven by a Reward effect on trials where the Task and Response either both switched, or both repeated, i.e. Task-Response congruent trials, with no Reward effect on Task-Response incongruent trials. This was confirmed by post-hoc tests on beta-values extracted from the anatomical (AAL) left putamen (see methods). This analysis revealed, on trials when the task repeated, a larger reward-related BOLD response during congruent (response repeat) vs. incongruent (response switch) trials (Reward x Response: F(1, 26) = 4.341, p = 0.047). This Reward x Response effect on trials when the task switched did not reach significance (F(1, 26) = 2.231, p = 0.147). Further disentangling these effects, there was a significant Reward effect on congruent repeat trials (when both the task and the response repeated: F(1, 26) = 8.5, p = 0.007) and on congruent switch trials (where both the task and response switched: F(1, 26) = 5.218, p = 0.031).

By contrast, this 3-way interaction effect (Reward x Task x Response) in the left putamen was not significant during the aPFC stimulation run (Fig. 4b - grey bars), consistent with an inhibitory effect of aPFC TMS on striatal activity. Stimulation of the aPFC did not result in any other significant changes in BOLD signal elsewhere in the brain as a function of the interaction between Reward, Task switching and Response switching (Supplementary Figure S1).

To determine the anatomical specificity of this TMS effect at the level of the striatum, a direct comparison between the beta-values in the anatomically defined left putamen and the left caudate nucleus during the integration between Reward, Task switching, and Response switching was performed. This revealed that the TMS (aPFC _BASE-STIM_) effect in the putamen was anatomically specific: as evidenced by a significant ROI (left putamen vs. left caudate nucleus) x Stimulation (aPFC_BASE-STIM_) x Reward x Task x Response interaction: F(1, 26) = 6.533, p = 0.017, η_p_^2^ = 0.201.

To determine the anatomical specificity of this TMS effect at the level of the cortex, we assessed whether the reduction in BOLD signal related to the 3-way interaction between Reward, Task and Response during the aPFC session was statistically different from the stimulation vs. baseline effect during the other two (i.e. dlPFC and PMC) sessions (Supplementary Information). This analysis revealed one significant cluster in the left putamen: P_SVC_FWE_ = 0.0248, t = 3.92, z = 3.83, peak x, y, z = −26, −8, 12) (Supplementary Figure S3). This finding shows that the effect of aPFC stimulation on task-related processing in the striatum was not likely related to non-specific effects such as the sensation of cTBS.

In summary, stimulation of the aPFC modulated activity in the posterior portion of the putamen as a function of the interaction between Reward, Task switching and Response switching (P_SVC_FWE_ = 0.020). This effect was anatomically as well as functionally specific, both at the level of the cortex and the striatum.

### Behavior

#### General task effects, irrespective of TMS

A detailed description of the behavioral effects across all three sessions can be found in the Supplementary Information, Supplementary Figure S4. In summary, across sessions, we observed a main effect of Reward, Task switching, and Response switching. In addition, we observed an effect of Reward on Task switching, and between Task switching and Response switching.

#### No effects of TMS on behavior

We did not observe any significant main effects of stimulation, irrespective of task conditions (in response times or error rates: F(1, 26) <1. None of the main effects (i.e. of Reward, Task or Response; Supplementary Information) were modulated by cTBS (stimulation vs. baseline) for any of the stimulation sites: response times and error rates all F(1, 26) <3.612, all p > 0.05. Finally, there was no effect of stimulation on the Reward x Task x Response effect for any of the stimulation sites (in response times and error rates: all F(1, 26) <2.316, all p > 0.1).

## Discussion

Functionally homogenous regions of the PFC and striatum are anatomically organized in parallel cortico-striatal circuits, connecting – for example – the anterior PFC and the anterior caudate nucleus as part of a reward circuit, and the motor cortices with the putamen as part of an action circuit. Moreover, the reward, cognition and action circuits are organized in an anterior to posterior and ventral to dorsal gradient, both in the frontal cortex and the striatum^3,11,12^. In the current study we aimed to assess these cortico-striatal circuits by asking whether task-related processing in the striatum is under control of the functionally homogenous region of the prefrontal cortex. For example, we aimed to assess whether reward-related processing in the caudate nucleus is under control of processing in the anterior PFC. To assess these hypothesized cortico-striatal causal interactions, we employed an offline TMS protocol aimed at decreasing neural signaling. In a three-session baseline-controlled counterbalanced within-subject crossover design, cTBS was applied over three distinct cortical sites, associated with motivational reward processing (anterior PFC), cognitive control (dorsolateral PFC) and action control (premotor cortex). Immediately following the application of cTBS, participants performed a rewarded task-switching paradigm in the MRI scanner. The paradigm tapped into all three cognitive domains: reward processing, task switching (cognitive control) and response switching (action control), allowing the assessment of task-related processing in distinct sub regions of the striatum (the caudate nucleus and the putamen).

We first assessed whether stimulation of the aPFC (compared with baseline) altered reward-related processing in the caudate nucleus. To correct for the comparison between two search volumes (caudate nucleus and putamen), we considered an effect P_SVC-FWE_ < 0.025 as significant. We observed a marginal decrease (P_SVC-FWE_ = 0.040) in the reward-related BOLD response in the striatum after aPFC stimulation compared with baseline. However, this effect did not reach significance according to our statistical threshold. Accordingly, we question its reproducibility, particularly given the relatively low physiological plausibility of the effect (with the cluster extending into the cerebrospinal fluid) and the lateralization to the right, non-stimulated hemisphere.

Next, we tested whether information from the reward circuit (i.e. the aPFC) could be transferred to another cortico-striatal circuit. We observed that stimulation of the aPFC altered processing in the putamen, the sub-region of the striatum that is part of the action circuit, but only when assessed as a function of the interaction between reward, cognitive control and action control. Specifically, aPFC stimulation elicited a reduction in task-related BOLD response (compared with the aPFC baseline fMRI run). Importantly, we also found that this aPFC_(BASE vs. STIM)_ effect was different from the effect in the dlPFC_(BASE vs. STIM)_ and PMC_(BASE vs. STIM)_ sessions. This is an important finding, because it shows that the effect of aPFC stimulation on task-related processing in the striatum was not likely related to non-specific effects such as the sensation of cTBS. These findings provide causal evidence in humans that stimulation of the aPFC causes a change in task-related processing in the striatum and, moreover, show that such distally induced task-related modulation is not restricted to the targeted cortico-striatal circuit (reward), but that modulated processing in the reward circuit can alter processing in another circuit (i.e. in the action circuit). Previous work has shown that cTBS over the aPFC can alter processing in regions distant from the site of stimulation^20^. However, in that study perfusion-MRI, instead of BOLD was used. In addition, the effects of aPFC stimulation were observed in the amygdala during the processing of emotional stimuli. We show here that the more commonly used BOLD-fMRI technique can be used successfully to assess cTBS-induced changes in task-related striatal processing.

The current study does not directly address the mechanisms by which aPFC stimulation affects processing in the putamen. However, based on previous work, two potential routes are most plausible. First, signals from the aPFC could reach the putamen via direct cortico-striatal connections^10–12,26^. Second, the signal from the aPFC could be conveyed to the putamen via spiraling dopaminergic connections between the striatum and the midbrain, i.e. striatal-nigro-striatal (SNS) connections^8,9,31–33^. Although results are largely consistent across species, it should be noted that these theories are derived primarily from work with non-human primates or rodents.

The anatomical specificity of the effects at the level of the cortex should be interpreted with caution. It cannot be argued that specifically aPFC stimulation, and not stimulation of the other two regions, can affect the integration between reward, cognition and action in the putamen. For this claim to hold, it would be necessary to show that stimulation of the dlPFC or PMC was effective. The absence of any task-related effects on striatal processing after stimulation of the dlPFC or PMC in this study thus precludes this claim. The null-effect following stimulation of the dlPFC and PMC was unexpected, especially considering previous work showing that stimulation of the dlPFC and the motor cortex can alter processing in the striatum^15,17,18^. However, contrary to those other studies, the current study is the first to assess the effect of cTBS on task-related processing in the striatum. Although Ko and colleagues^15^ applied cTBS over the prefrontal cortex, they did not assess task-related processing, nor did they assess effects with BOLD-fMRI. While two other studies did observe effects of cortical TMS on task-related BOLD-fMRI in the striatum^17,18^, those studies did not stimulate the PMC, but the primary motor cortex, using a different TMS protocol (rTMS: 1–10 Hz instead of cTBS).

One explanation of the null effect after dlPFC and PMC stimulation is that - irrespective of TMS - we did not observe an effect of task switching or response switching in the striatum. This was true both across all sessions, and during the relevant baseline session (e.g. the baseline dlPFC session for the effect of task switching). The logic of testing whether stimulation of a cortical region will reduce striatal BOLD response does not hold unless there is a measurable BOLD response in the striatum during baseline. Alternatively, it is conceivable that the cortical regions we stimulated do not have a (causal) role in switching between tasks (dlPFC) or response buttons (PMC). This is consistent with the distance between the peak of the task-related fMRI activity (white circles in Fig. 3d–f) and the stimulation sites (arrow in Fig. 3d–f**)**. Stimulation was targeted based on fMRI coordinates from a previous study^22^. However, the coordinates functionally activated in the present study, both for task-switching (dlPFC) and response switching (PMC), differed from the previous sample to the extent that no task-related activation actually overlapped with the coordinates at which TMS was applied. Although the exact spatial resolution of TMS is highly dependent on several factors, a resolution in the range of millimeters up to several centimeters has been suggested^34^. In any case, the field induced by TMS declines rapidly with distance from the coil^35^. In the current study, this implies that we might have failed to stimulate the task-relevant circuits for the dlPFC and PMC but not for the aPFC sessions. More specifically, a meta-analysis of fMRI coordinates associated with task switching has identified the inferior frontal junction as a key node^36^. The region reported in the meta-analysis (x, y, z coordinates: −40, 4, 30) overlaps with a cluster activated by the task-switching contrast in the current study (x, y, z coordinates: −40, 4, 30). This same cluster in the inferior frontal gyrus has been observed in two previous studies using the same paradigm (x, y, z coordinates: −52, 6−, 34^24^; x, y, z coordinates: −48, 12, 28^25^). In combination, this suggests that the main effect of task switching in the inferior frontal gyrus observed in the current study was most likely a reliable effect, and that the dlPFC region we stimulated was too anterior to target the cortico-striatal circuitry crucial for task switching.

In terms of response switching, previous fMRI studies assessing effects of response switching embedded in a task-switching paradigm^37,38^, reported effects more posterior (y = 0) than our stimulation coordinate (y = 10). Interestingly, both studies reported peak coordinates that are located within the parietal cluster we report, suggesting that this may have been a more successful target for cTBS in the current study.

We previously reported modulation of the BOLD response in dorsal parts of the striatum during the integration between reward and task switching^22^. This interaction was not observed in the current dataset. An important feature of the previous work^22^ however was that those effects were revealed exclusively as a function of inter-individual variance in the dopamine transporter (*DAT1*) genotype^22,25^.

The current study was designed to modulate processing in the striatum. In keeping with this hypothesis, we showed that stimulation of the aPFC modulates BOLD responses in the striatum. However it did not induce any behavioral changes, which is not uncommon with offline TMS^17,39^. The absence of a behavioral effect precludes us from making any claims about whether stimulation of the aPFC and the consequent effects on the striatum were behaviorally relevant. On the other hand, it simplifies the interpretation of the observed BOLD changes. If stimulation had also changed behavior, this in itself would be expected to alter task-related signal in the striatum, complicating interpretation. In the absence of such effects, the observed changes in brain activity can be straightforwardly attributed to a causal effect of cortical TMS.

Several neuropsychiatric/neurological disorders, such as substance use disorder, obsessive-compulsive disorder, schizophrenia and Parkinson’s disease are accompanied by altered processing in cortico-striatal circuits for reviews see refs^40,41^. In addition, patients with Parkinson’s disease, obsessive-compulsive disorder, and depressive disorder can benefit from deep brain stimulation of subcortical regions such as the striatum^42–44^. Alternatively, TMS provides a potentially less-invasive method to target subcortical structures. In fact, TMS is currently being used as an FDA approved treatment for major depressive disorder and has been suggested as a potential treatment for patients suffering from schizophrenia^45^. The current work adds to the knowledge required for a better understanding of ways by which subcortical circuits can be targeted effectively via non-invasive cortical stimulation. We show that stimulation with cTBS, which is well tolerated over the anterior prefrontal cortex, can alter processing in the striatum.

## Electronic supplementary material

Supplementary information
