# Supplementary material for: Controlling striatal function via anterior frontal cortex stimulation
Source: Sci Rep. 2018 Feb 19;8:3312. doi: 10.1038/s41598-018-21346-5 (PMC5818614; doi:10.1038/s41598-018-21346-5)
Supplement: Supplementary file 1 — Supplementary information [file 41598_2018_21346_MOESM1_ESM.pdf]

Supplementary Information

Title:

Controlling striatal function via anterior frontal cortex stimulation

Authors:

Mieke van Holstein* [1,2], Monja I. Froböse [1], Jacinta O'Shea [1,3], Esther Aarts [1], Roshan Cools [1,4]

Affiliations:

[1] Radboud University, Donders Institute for Brain, Cognition and Behavior, Nijmegen, The Netherlands

[2] Department of Psychology and Brain Research Center, University of British Columbia, Vancouver, BC, Canada

[3] Wellcome Centre for Integrative Neuroimaging (WIN), Oxford Centre for Functional MRI of the Brain (FMRIB), Nuffield Department of Clinical Neurosciences, University of Oxford, John Radcliffe Hospital, Headington, Oxford OX3 9DU, UK.

[4] Radboud University Medical Center, Department of Psychiatry, Nijmegen, The Netherlands

* Corresponding author:

Mieke van Holstein, University of British Columbia, 2211 Wesbrook Mall, Vancouver, BC, V6T 1Z3, Canada; email address: miekevanholstein@gmail.com

Supplementary methods

Participants

One participant was excluded due to a contra-indication for MRI, one participant's session was discontinued due to dizziness during MRI, one participant was excluded due to technical TMS problems, and one participant due to technical MRI problems. All 27 included participants had normal or corrected-to-normal vision, were right-handed and pre-screened for claustrophobia, psychiatric, neurological, and vascular disorders, drug and medication use, alcohol consumption and smoking behavior, as well as any contraindications for TMS and MRI.

Paradigm

Practice blocks

The first practice block (24 trials), which was only administered during the intake session, included only task-switching to familiarize participants with the alternation between tasks (arrow vs. word). During this block, the task (i.e. whether to respond to the arrow or the word) alternated unpredictably from trial to trial without any reward cues, and the feedback on each trial was either "correct" or "incorrect". During the intake session and at the start of each experimental session, participants completed a second practice block that was exactly the same as the actual paradigm described in the legend of **figure 2**, only shorter (i.e. 24 trials). Finally, a third block (32 trials) without reward or feedback was administered in the scanner immediately before the actual paradigm started, and during the intake session (see main text: **intake session**). This third block was used to determine each individual's response window. We calculated the average response times on four trial types (arrow, word x task-switch, task-repeat), during the third practice block. These response times were set as the response deadline during the subsequent run. This was done to account for inter-individual and inter-run differences in response speed and subsequent task difficulty.

Transcranial Magnetic Stimulation (TMS) procedure

Selection and targeting of stimulation sites

To determine the TMS coil positioning for each individual and each brain region, each participant's structural scan was coregistered to the standard SPM8 T1 template (Montréal Neurological Institute; MNI) and segmented using a unified segmentation procedure[27]. This procedure resulted in a set of inverse parameters allowing the conversion of the stimulation targets in group mean MNI coordinates into individuals' native anatomical space. Next, the MNI coordinates for each cortical stimulation site were projected onto each individual's structural scan using a frameless stereotactic neuronavigation system (Localite, Sankt Augustin, Germany). The TMS coil was then placed on the scalp overlying the target coordinates (aPFC, dlPFC, PMC) using the Localite software.

During the intake session, participants were familiarized with the sensation of cTBS over each of these regions. Thirty-nine participants started the intake session. Any participant who reported - or showed - signs of discomfort during this part of the intake session was excluded from further participation. This resulted in the exclusion of eight participants: six participants due to discomfort during stimulation over the aPFC and two due to a more general feeling of discomfort during this part of the intake. As a result, 31 participants started the main experiment (see **participants**).

Continuous Theta Burst Stimulation (cTBS) protocol

During the determination of the active motor threshold (aMT), participants rested their right hand on a pillow while squeezing a small roll of tape with a pincer grip at 20% of their maximum strength, contracting their first dorsal interosseous FDI muscle continuously. The aMT was defined as the lowest stimulation intensity over the contralateral motor cortex that elicited reproducible MEPs (in at least 5 out of 10 successive stimulations). The aMT was 24%-37% (mean 30.44%, SD 3.61) of the maximum stimulator output.

Magnetic Resonance Imaging (MRI) procedure

Preprocessing of task-related fMRI data

Prior to standard preprocessing, realignment was performed using the estimated head motion parameters (least-squares approach, 6 parameters) for the images with the shortest echo, which were applied to echo images for each excitation. The images of all sessions were aligned to the shortest echo of each session, and to the first session. After spatial realignment, the four echo images were combined using echo summation. The combined images were slice-time corrected to the middle slice and segmented using a unified segmentation procedure[27]. The bias corrected T1 image was coregistered to the mean functional image and the transformation matrix from the segmentation procedure was used for normalization to a standard template (MNI). Normalized images were smoothed using an 8 mm full-width half maximum kernel. A study– specific T1 template was generated from an average of all co-registered and normalized T1 images to display the results, using MRIcron software.

Statistical analysis of fMRI data

First-level analysis

We automatically included any participant with <3mm (one voxel size) of head motion in either direction (N=22). For the 5 participants who moved more than 3mm (but never more than 2 voxel sizes), we made sure movement was gradual, which is easier to correct for. Two participants did show a peak of excessive movement in one of their 6 fMRI runs. We attempted to account for residual head motion by including –for all

participants - the six original head motion parameters (3 translation, 3 rotation), their first derivative and the square of the original and first derivative in the model, resulting in 24 motion nuisance regressors[30]. Before including a participant with more than 3mm of head motion, we assessed the activation maps of the affected fMRI run to make sure there were no residual motion artifacts in the individual activation maps. Finally, we repeated the analysis of our primary result to confirm that the 4-way interaction of $aPFC_{BASE-STIM}$ x Reward $_{HIGH\ vs.\ LOW}$ x Task $_{SWITCH\ vs.\ REPEAT}$ x Response $_{SWITCH\ vs.\ REPEAT}$ in the left putamen remained significant after the exclusion of the two participants with excessive head motion.

In addition, we used the mean signal from the white matter and CSF to account for movement-related intensity changes[31]. Finally, a high-pass filter (128s) was used to remove low-frequency signals (e.g. scanner drifts) and an AR(1) model was applied to adjust for serial correlations in the data. Microtime onsets were adjusted to account for the earlier mentioned slice time correction.

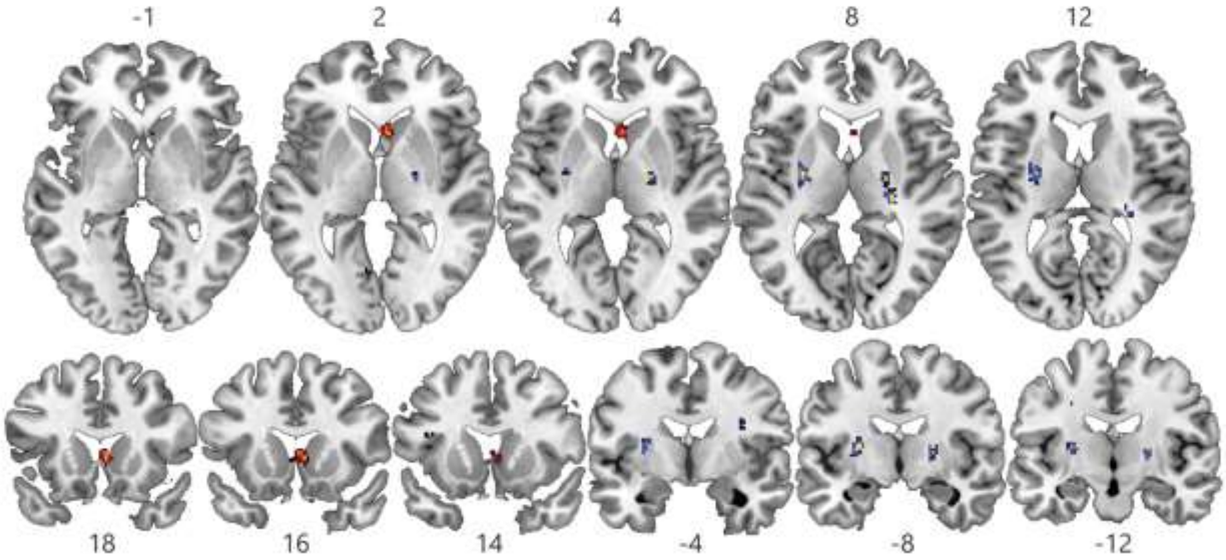

Supplementary figure S1 | Whole-brain maps for the effect of aPFC stimulation on the main effect of Reward (in red) and the interaction between Reward, Task switching and Response switching (blue). Whole-brain maps across the axial (dorsal to ventral) and coronal (anterior to posterior) plane are shown for the effects in figure 4. The effect of aPFC stimulation on the main effect of Reward is shown in red. The effect of aPFC stimulation on the interaction between Reward, Task switching and Response switching in blue. Note that the results are displayed at a low threshold ($P_{UNC} < 0.001$, $t > 3.14$), but that statistical significance (FWE-corrected) of the results was assessed in two anatomically defined regions of interest (i.e. restricted to the grey matter of the bilateral caudate nucleus and bilateral putamen).

Functional and anatomical specificity: Supplementary methods and results

Functional specificity

To assess functional specificity, we tested whether TMS over the aPFC altered processing in the caudate nucleus exclusively as a function of Reward processing. In other words, we anticipated that TMS over the aPFC would not alter processing in the caudate nucleus as a function of Task switching or Response switching.

In addition, we assessed whether TMS over the aPFC altered processing in the putamen exclusively as a function of the interaction between Reward, Task switching and Response switching, and not as a function of any other effects (e.g. the effect of Reward, Task switching, *or* Response switching, or the interaction between Reward and Task switching).

The effects of aPFC stimulation on reward-related processing in the caudate nucleus and the effects of aPFC stimulation on the interaction between Reward, Task switching and Response switching in the putamen were functionally specific: aPFC stimulation did not decrease striatal processing as a function of Task switching (caudate nucleus: k = 0, putamen: P_{SVC_-FWE} = 0.165, k = 2) or Response switching (caudate nucleus: P_{SVC_-FWE} = 0.150 k = 3, putamen: k = 0). In addition, the effects of aPFC stimulation did not decrease striatal processing as a function of the interaction between Reward and Task switching.

Anatomical specificity at the level of the striatum

We assessed quantitatively the anatomical specificity of an effect in a region. For example, we aimed to assess whether the effect of aPFC stimulation on the effect in the left putamen (**figure 4b**) was different from the same effect in the left caudate nucleus. To avoid double dipping [32], we derived beta values from an independent anatomical ROI (i.e. independent from the activated cluster). Values were entered into a repeated measures GLM in SPSS with the factors stimulation ($aPFC_{STIM\ vs.\ BASE}$), ROI (caudate nucleus vs. putamen) and either 1) Reward $_{(HIGH\ VS.\ LOW)}$ (time-locked to the reward cue) or 2) Reward $_{(HIGH\ VS.\ LOW)}$, Task $_{(SWITCH\ VS.\ REPEAT)}$ and Response $_{(SWITCH\ VS.\ REPEAT)}$ (all time-locked to the target).

A direct comparison between the effect of Reward in the right caudate nucleus and the effect of reward in the right putamen revealed that the (trending) effect in the right caudate nucleus was specific to the caudate nucleus: ROI (right caudate nucleus vs. right putamen) x Stimulation ($aPFC_{BASE-STIM}$) x Reward: $F(1,26) = 4.937$, $p = 0.035$, $\eta_p^2 = 0.159$

The effect in the putamen during the integration of Reward, Task and Response was anatomically specific: these results are discussed in the main text.

Anatomical specificity at the level of the cortex

To test whether the effect of aPFC stimulation on Reward-processing in the caudate nucleus was anatomically specific at the level of the cortex, we submitted the data from each session for the factor Reward to a GLM with the additional factor Site (aPFC, dlPFC, PMC).

This allowed us to assess whether the (marginal) effect of aPFC stimulation $_{(BASE-STIM)}$ on reward-related processing in the caudate nucleus (**figure 4a**) was different during the aPFC session compared to the other two session. This interaction test (Stimulation x Site (i.e. aPFC $_{BASE - STIM}$ > dlPFC $_{BASE - STIM}$ = PMC $_{BASE - STIM}$) x Reward) revealed no significant voxels in the whole-brain or the after applying a SVC in the striatum.

To test whether the effect of aPFC stimulation on the interaction between Reward, Task and Response was significantly different compared to the data from the dlPFC ($_{BASE - STIM}$) and PMC ($_{BASE - STIM}$) sessions, we submitted the data from each session for the Reward x Task x Response contrast to a GLM with the additional factor Site (aPFC, dlPFC, PMC), resulting in the following interaction test: Stimulation x Site (i.e. aPFC $_{BASE - STIM}$ > dlPFC $_{BASE - STIM}$ = PMC $_{BASE - STIM}$) x Reward x Task x Response. This analysis revealed one significant cluster in the left putamen: $P_{SVC-FWE} = 0.0248$, $t = 3.92$, $z = 3.83$, peak x, y, z = -26, -8, 12). The whole-brain map for this interaction is shown in **figure S3a** at a threshold of $P_{UNCORRECTED} < 0.001$. To visualize this interaction we extracted the beta values as described elsewhere (see **order effects**). For an unbiased representation of the data, we extracted the beta values from the left anatomically defined putamen. We plotted the interaction between Reward, Task switching and Response switching on the Y-axis of **figure S3b**, separately for each stimulation site (aPFC, dlPFC, PMC) and for the stimulation fMRI vs. baseline fMRI run.

Order effects: Methods

We aimed to assess whether the results in the caudate nucleus (main effect of reward) and putamen (the Reward x Task switching x Response switching interaction), were dependent on the order in which participants performed the baseline and stimulation fMRI run (Stimulation order), i.e. stimulation fMRI followed by a baseline fMRI (N=13) or the opposite arrangement (N=14) (**figure 1**). We reasoned that any residual effect of the inhibitory TMS protocol in those who performed the stimulation fMRI run first, would be evident in a reduced effect during the baseline fMRI run in this group of participants. In addition, we aimed to assess whether we could find any evidence to suggest that the order of the stimulation Site (Site order), i.e. on which day the aPFC stimulation took place (i.e. during the 1st, 2nd or 3rd session), had an effect on the Reward effect or on the interaction between Reward, Task switching, and Response switching.

We extracted the beta values during the Reward cue (high and low Reward) from the cluster in the right caudate nucleus as presented in **figure 4a**. Next, using SPPS software (IBM SPSS Statistics 23), we performed a repeated measures GLM with the within-subject factors Stimulation (aPFC stimulation vs. aPFC baseline), and Reward (high vs. low) and the between subject factors Stimulation order and Site order (as described above).

From the cluster in the left putamen, as presented in **figure 4a**, we extracted the beta-values during target for the interaction between Reward, Task switching and Response switching and entered these variables as well as Stimulation (aPFC stimulation vs. aPFC baseline) as within-subject factors. We added the between subject factors Stimulation order and Site order (as described above).

In addition, because we observed a Site (aPFC, dlPFC, PMC) x Stimulation (stimulation vs. baseline) x Reward x Task x Response interaction in the putamen (**figure S3a**), we also assessed whether that interaction varied either as a function of Stimulation order or Site order. Because the voxels from which the beta values were extracted were based on the interaction between $aPFC_{BASE-STIM}$, we assessed these effects in an unbiased region: the anatomically defined left putamen.

Order effects: Results

The effect of aPFC stimulation on Reward processing in the caudate nucleus

We did not find any evidence to suggest that the effect of aPFC stimulation vs. aPFC baseline on the (trending) Reward-related signal in the caudate nucleus (**figure 4a**) was different in those participants who started the session with the baseline fMRI run (**figure S2a** dark red bars: baseline followed by stimulation) compared with those who started the session with cTBS followed by the stimulation fMRI run (**figure S2a** light red bars: simulation followed by baseline). More specifically, the Reward x Stimulation ($aPFC_{BASE-STIM}$) x Baseline order interaction was not significant ($F(1,21)<1$. In addition, there was no evidence that the effect of aPFC stimulation

vs. aPFC baseline on the Reward-related signal in the caudate nucleus was dependent on the session number (1st, 2nd or 3rd) in which the aPFC was stimulated ($F(2,21) = 2.351$, $p > 0.05$.

The effect of aPFC stimulation on the integration between Reward, Task and Response in the putamen

Inspection of **figure S2b** suggests that the effect of aPFC stimulation vs. baseline on the integration between Reward, Task switching and Response switching (**figure 4** – in blue) was smaller in participants that started the session with the baseline fMRI run (**figure S2b** darker blue bars), compared with the other group (**figure S2b** lighter blue bars). However, this effect was not significant: The effect of aPFC stimulation vs. aPFC baseline on the integration between Reward, Task, and Response in the putamen was not different for those participants who started the session with the baseline fMRI run (**figure S2b** dark blue bars: baseline followed by stimulation) compared with those who started the session with cTBS followed by the stimulation fMRI run (**figure S2b** light blue bars: stimulation followed by baseline). More specifically, the Stimulation ($aPFC_{BASE-STIM}$) x Reward x Task x Response x Baseline order interaction was not significant ($F(1,21) = 3.672$, $p > 0.05$).

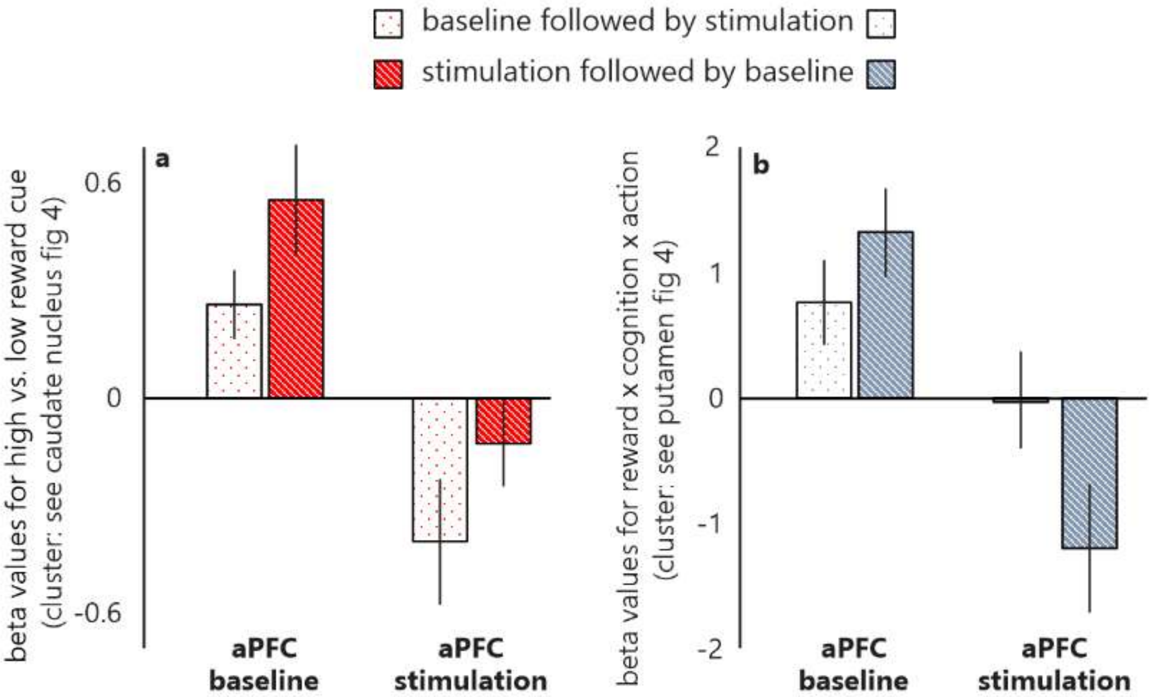

Supplementary figure S2 | Effects of order (baseline vs. stimulation fMRI run)

Plots of beta-values extracted from **a)** the right caudate nucleus cluster (see figure 4a) for the contrast high vs. low Reward and for **b)** the left putamen cluster (see figure 4b) for the contrast high vs. low Reward x Task switching (switch vs. repeat) x Response switching (switch vs. repeat). Results are shown separately for participants who performed the baseline fMRI run prior to the stimulation fMRI run (light bars) and those who performed the stimulation fMRI run before the baseline fMRI run (dark bars).

Moreover, the observed (not significant) pattern was opposite to what we would expect if the cTBS stimulation had carried over to the baseline fMRI run. If the 90 minutes between cTBS stimulation and the cTBS baseline fMRI run had not been sufficient, we would expect to see the opposite pattern from the one observed here: I.e., we would expect a smaller difference between the aPFC stimulation and the subsequent aPFC baseline session in those participants who started with the stimulation run (the lighter bars in **figure S2b**).

In addition, there was no evidence that the effect of aPFC stimulation vs. aPFC baseline on the Reward, Task, Response-related signal in the putamen (**figure 4b**) was dependent on the session number (1st, 2nd or 3rd) in which the aPFC was stimulated (Stimulation (aPFC $_{BASE-STIM}$ x Reward x Task x Response x Site order: $F(2, 21) < 1$).

Finally, we repeated the analyses and included data from all sites (aPFC, dlPFC and PMC), to assess whether we could find evidence that the Site x Stimulation x Reward x Task x Response interaction in the putamen (**figure S3**) was modulated by either Stimulation order or Site order. We did not find evidence for these effects (both F's < 1).

Inspection of **figure S2b** suggests an effect in the putamen as a function of the interaction between Reward, Task switching and Response switching during the baseline fMRI runs of the aPFC and PMC session, but not during the dlPFC session. Indeed, a direct contrast between the baseline aPFC and the baseline dlPFC session (grey bars) revealed that the BOLD signal in the left putamen was lower during the dlPFC baseline session (Site (aPFC $_{BASE}$ vs. dlPFC $_{BASE}$) x Reward x Task x Response: $P_{FWE-SVC} = 0.019$), but that there was no evidence of a difference between the baseline runs during the aPFC and PMC sessions (Site (aPFC $_{BASE}$ vs. PMC $_{BASE}$) x Reward x Task x Response: $P_{FWE-SVC} > 0.05$). To assess directly in the left putamen whether the effect in **figure S2** can be explained by a difference in BOLD response during the baseline sessions, we performed a direct test of the Reward x Task x Response x Stimulation interaction between the aPFC and PMC session (i.e. sessions with comparable baselines). This revealed a significant cluster in the left anatomical putamen (aPFC $_{BASE-STIM}$ > PMC $_{BASE-STIM}$ x Reward x Task x Response: $P_{FWE-SVC} = 0.026$, k = 26, t = 3.63, z = 3.55, x, y, z peak = -26, -8, 12). This analysis confirms that the significant 4-way interaction, reported in the main text, i.e. aPFC $_{BASE-STIM}$ > dlPFC $_{BASE-STIM}$ = PMC $_{BASE-STIM}$) x Reward x Task x Response, was not driven by the dlPFC session.

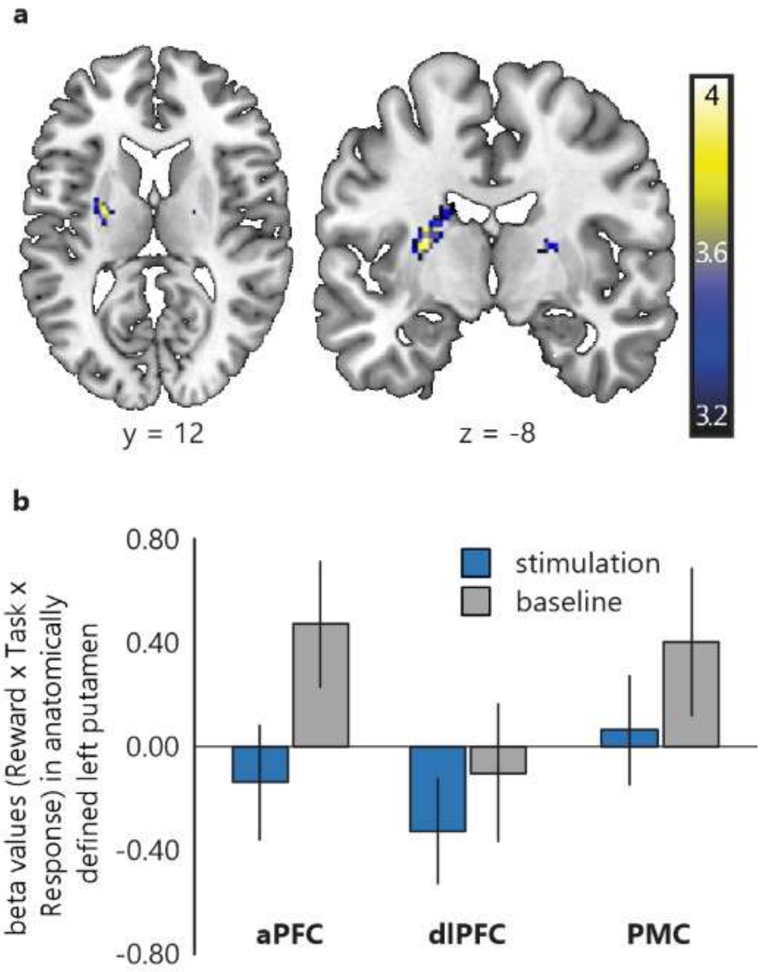

Supplementary figure S3 | Effect of aPFC vs. dlPFC and PMC stimulation (vs. baseline) for the interaction between Reward, Task switching and Response switching
a) Brain maps for the interaction effect of cTBS (stimulation vs. baseline) x Site (aPFC, dlPFC, PMC) x Reward x Task switching x Response switching (shown at $P_{UNC} < 0.001$, note that the cluster in the left putamen is significant at $P_{FWE\text{-}SVC} = 0.026$). Color scales reflect t-values. **b)** Plots of the beta-values extracted from the anatomically defined left putamen for the same contrast as in figure S3a.

Differences in the baseline run do not account for the effect of aPFC (vs. dlPFC and PMC) stimulation in the putamen

When inspecting the two sessions with equal baselines in **figure S3b**, it can be seen that the effect of aPFC $_{\text{BASE-STIM}}$ vs. the PMC $_{\text{BASE-STIM}}$ is driven by a combination of a numerical increase in the Reward x Task x Response –related BOLD response during the aPFC baseline fMRI run and a numerical decrease in the Reward x Task x Response –related BOLD response during the aPFC stimulation fMRI run. This was confirmed by a post-hoc test: direct comparison of the aPFC stimulation vs. PMC stimulation runs or the aPFC baseline vs. PMC baseline runs did not result in a significant cluster in the putamen. Combined, these effects resulted in a significant effect. In the absence of a baseline session, we would not have been able to detect this effect..

Thus, the inclusion of the baseline fMRI runs in our design has enabled us to take into account variation in task-related BOLD response within the same individuals across different days. This enabled us to increase the sensitivity of our measurements, quantifying inter-session variance that would otherwise have been undetectable.

Behavioral data

Methods: Statistical analysis of behavioral data

The first trial of each block, trials with extremely fast responses (<100ms), and trials on which participants failed to respond were excluded from analysis. In addition, trials on which the response was incorrect were excluded from RT analyses. The RT and error rate data violated a normal distribution, which was not resolved after log and arcsine transformations, respectively (Shapiro-Wilk $p < 0.05$). Therefore, effects that reached significance were submitted to a non-parametric Wilcoxon signed-rank test to assure that the repeated measures GLM did not reflect false-positives. We did not observe any false-positives.

Results: Behavioral effects across all sessions

Across sessions, participants responded faster on high reward trials (mean $\pm$ SE: 368.07 $\pm$ 5.69ms) compared with low reward trials (mean $\pm$ SE: 375.87 $\pm$ 6.33ms) (Reward: $F(1,26) = 30.930$, $p < 0.001$, $\eta_p^2 = 0.543$**; figure S4**). There was no main effect of reward in terms of error rates (Reward: $F(1,26) < 1$). In terms of task-switching performance, participants made more errors on task-switch trials (mean $\pm$ SE: 17% $\pm$ 1.6%) compared with task-repeat trials (mean $\pm$ SE: 13.7 $\pm$ 1.2%) (Task switching: $F(1,26) = 25.527$, $p < 0.001$, $\eta_p^2 = 0.495$; **figure S4**), but showed no main effect of task switching in terms of response times (Task switching: $F(1,26) = 2.146$, $p = 1.55$). Finally, participants responded more slowly (mean $\pm$ SE: 374.26 $\pm$ 6.18ms) and made more errors (mean $\pm$ SE: 17.2 $\pm$ 1.5%) when the same response had to be repeated compared with trials on which the response switched (mean $\pm$ SE: 369.68 $\pm$ 6.14ms and 13.5 $\pm$ 1.3% respectively) (Response switching in terms of response times: $F(1,26) = 8.454$, $p = 0.007$, $\eta_p^2 = 0.245$), and error rates $F(1,26) = 21.333$, $p < 0.001$, $\eta_p^2 = 0.451$; **figure S4**).

Across sessions, participants exhibited a significant effect of reward on task switching in terms of response times (Reward x Task: $F(1,26) = 40.691$, $p < 0.001$, $\eta_p^2 = 0.61$; **figure S4**), but not in terms of error rates (Reward x Task: $F(1,26) < 1$). Breaking down this effect in the response times revealed that participants exhibited a task-switch benefit (i.e. task repeat (mean $\pm$ SE: 377.73 $\pm$ 6.23ms) – task switch (mean $\pm$ SE: 374.00 $\pm$ 6.51ms)) on low reward trials ($F(1,26) = 7.805$, $p = 0.010$, $\eta_p^2 = 0.231$) and a switch cost (i.e. task switch (mean $\pm$ SE: 371.57 $\pm$ 6.28ms) – task repeat (mean $\pm$ SE: 364.58 $\pm$ 5.71ms) performance) on high reward trials ($F(1,26) = 23.305$, $p < 0.001$, $\eta_p^2 = 0.473$). In addition, in terms of errors rates, we observed a larger task-switch cost on response repetition trials (task switch: mean $\pm$ SE: 19.7% $\pm$ 1.8%; task repeat: mean $\pm$ SE: 14.6% $\pm$ 1.4%; main effect of task: $F(1,26) = 25.910$, $p < 0.001$, $\eta_p^2 = 0.499$) than on response switch trials (task switch: mean $\pm$ SE: 14.3% $\pm$ 1.5%; task repeat: mean $\pm$ SE: 12.8% $\pm$ 1.2%; main effect of task: $F(1,26) = 4.266$, $p = 0.049$, $\eta_p^2 = 0.141$; **figure S4**). This was evidenced by a significant Task x Response interaction (error rates: $F(1,26) = 9.489$, $p = 0.005$, $\eta_p^2 = 0.267$; response times: $F(1,26) <1$). There was no Reward x Task x Response interaction ($F(1,26) < 1$ for response times or error rates).

Discussion of behavioral data

A number of studies have reported that task-switching and response switching are not independent[1,2]. This is substantiated by our behavioral data. The task-switch cost observed was larger on response-repeat trials, in agreement with previous reports[1,2]. In addition, we observed a behavioral response switch benefit: participants were faster and more accurate when responding on a response switch compared with a response repeat trial **(figure S4)**. When inspecting **figure S4**, it becomes clear that there was a considerable behavioral cost associated with trials on which the task switched, but the response remained the same. It appears that this increased error rate on response repeat/task switch trials is driving both the interaction and the main effect.

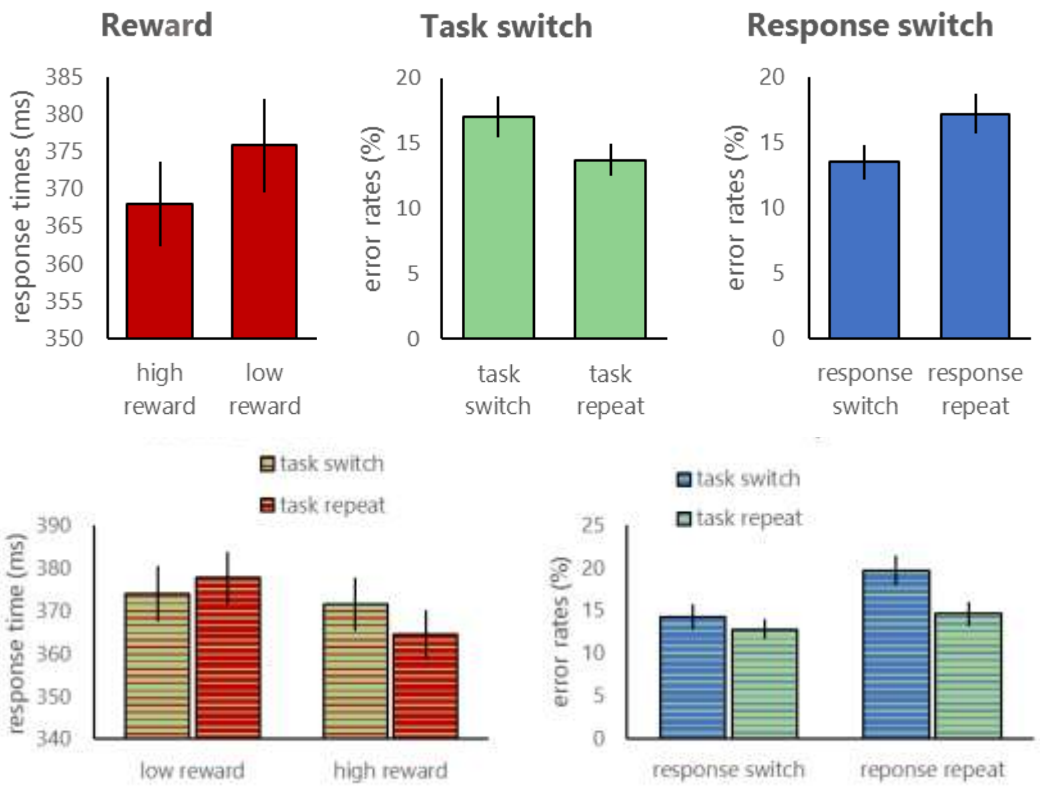

Supplementary figure S4 | Behavioral data across all sessions
Data are shown for **top:** the main effect of reward (response times), task switching (error rates) and response switching (error rates), and **bottom:** for the interaction between reward and task switching (Reward x Task; response times) and for the interaction between task switching and response switching (Task x Response; error rates). All effects are shown across all 6 runs, i.e. irrespective of TMS.

Supplementary references

1. Shook, S. K., Franz, E. A., Higginson, C. I., Wheelock, V. L. & Sigvardt, K. A. Dopamine dependency of cognitive switching and response repetition effects in Parkinson's patients. *Neuropsychologia* **43,** 1990–1999 (2005).

2. Stelzel, C., Basten, U. & Fiebach, C. J. Functional connectivity separates switching operations in the posterior lateral frontal cortex. *J. Cogn. Neurosci.* **23,** 3529–3539 (2011).
